# High expression of JC polyomavirus-encoded microRNAs in progressive multifocal leukoencephalopathy tissues and its repressive role in virus replication

**DOI:** 10.1371/journal.ppat.1008523

**Published:** 2020-04-23

**Authors:** Kenta Takahashi, Yuko Sato, Tsuyoshi Sekizuka, Makoto Kuroda, Tadaki Suzuki, Hideki Hasegawa, Harutaka Katano

**Affiliations:** 1 Department of Pathology, National Institute of Infectious Diseases, Shinjuku, Tokyo, Japan; 2 Pathogen Genomics Center, National Institute of Infectious Diseases, Shinjuku, Tokyo, Japan; Louisiana State University, UNITED STATES

## Abstract

JC polyomavirus (JCPyV, JCV) causes progressive multifocal leukoencephalopathy (PML) in immunocompromised hosts. JCPyV replicates in oligodendrocytes within the brain tissue of patients with PML. The JCPyV genome encodes a microRNA (miRNA) in the region encoding the large T antigen. JCPyV-encoded miRNA (miR-J1) has been detected in the tissue and cerebrospinal fluid samples of patients with PML; however, there are no reports describing the localization of polyomavirus-encoded miRNA in histological samples of patients with virus-associated diseases. In the present study, we detected high miR-J1 expression in the nuclei of JCPyV-infected cells in PML tissue samples via *in situ* hybridization. Additionally, *in situ* hybridization also revealed the expression of BK polyomavirus (BKPyV, BKV)-encoded miRNA in lesions of BKPyV-associated nephropathy. *In situ* hybridization for miR-J1-5p and -3p showed positive signals in 24/25 (96%) of PML tissues that were positive for JCPyV by immunohistochemistry. Higher copy numbers of miR-J1 were detected in PML tissues than in non-PML tissues by real-time reverse transcription PCR. Next generation sequencing showed that miR-J1-5p, a mature miRNA of primary miRNA, was predominant in the lesions compared with miR-J1-3p, another mature miRNA. Deletion or mutation of miR-J1 in recombinant JCPyV promoted the production of JCPyV-encoded proteins in cells transfected with JCPyV DNA, suggesting that polyomavirus-encoded miRNA may have a repressive role in viral replication in PML tissues. *In situ* hybridization for viral miRNA may be a useful diagnostic tool for PML.

## Author summary

JCPyV causes PML in immunocompromised hosts. In patients with PML, JCPyV replicates in the oligodendrocytes of brain tissue. A JCPyV-encoded miRNA, miR-J1, has been detected by real-time PCR in the brain tissue and cerebrospinal fluid of patients with PML; however, there are no reports describing the localization of polyomavirus-encoded miRNA in histological samples of patients with virus-associated diseases. In this study, *in situ* hybridization clearly showed that miR-J1 was highly expressed in the nuclei of JCPyV-infected cells within PML tissue samples. A high level of BKPyV-encoded miRNA expression was also found in BKPyV-associated nephropathy. The results of experiments with recombinant JCPyV demonstrate that a defect of miR-J1 promotes JCPyV replication in cells transfected with JCPyV DNA. The observed high expression of viral miRNA, which has a repressive role in virus replication, suggests an autoregulation mechanism in virus replication as well as the possibility that viral miRNA may be a new therapeutic tool for PML.

## Introduction

MicroRNAs (miRNAs) are short fragments of RNA with 20–22 mer length [1, 2]. Almost all animals encode miRNAs in their genomes and exhibit miRNA expression [1]. Many DNA viruses also encode their own miRNAs [3–5]. Virus-infected cells express viral miRNAs inside the cells and deliver them outside the cells [6, 7]. Thus, viral miRNA has been detected in the serum of patients with viral infections [8–10]. Viral miRNAs have various, and sometimes multiple, functions [3, 5, 11]. Like cellular miRNAs, viral miRNAs bind to host mRNAs and affect their translation [5, 11].

JC polyomavirus (JCPyV, JCV) is a human polyomavirus causing progressive multifocal leukoencephalopathy (PML) in immunocompromised hosts [12–16]. This virus is common among the general population. Most people are infected with JCPyV in childhood, and about 80% of adults are positive for anti-JCPyV antibodies [17, 18]. JCPyV is a small DNA virus with a 5-kbp genome that encodes the large T (LT) and small t antigens, VP1-3, and agnoprotein [19]. Lytic infection of oligodendrocytes by JCPyV induces demyelination of brain tissue, and PML lesions contain cells expressing these viral proteins [20, 21]. Immunohistochemistry, therefore, is a useful tool for the identification of virus-infected cells in PML tissues [22].

JCPyV encodes a primary miRNA (pri-miR-J1) in the LT-coding region of the viral genome but in opposed sense [23]. The pri-miR-J1 is processed into a precursor miRNA (pre-miR-J1) that produces two mature miRNAs, miR-J1-5p and -3p [23]. Real-time polymerase chain reaction (PCR) and northern blot analyses have detected both mature miRNAs of miR-J1, not only in the brain tissue and cerebrospinal fluid of patients with PML, but also in the blood and urine of patients without PML and of healthy individuals [23–25]. Thus, the amounts of miR-J1 in blood and urine are unrelated to the progression of PML. To date, there have been no reports describing the localization of polyomavirus-encoded miRNA in histological samples from patients with virus-associated diseases. Although functions of miR-J1 have been rarely reported [11], this miRNA has been indicated to have a repressive role in JCPyV replication [23].

BK polyomavirus (BKPyV, BKV) is another human polyomavirus that is associated with viral nephropathy [26, 27]. BKPyV also encodes miRNA in its genome, pri-miR-B1, which is processed into pre-miR-B1 and produces two mature miRNAs, miR-B1-5p and -3p; however, one of the mature miRNAs, miR-B1-3p, has the same sequence as miR-J1-3p [11, 23]. miR-B1 was detected in urine and serum samples from patients with BKPyV-associated nephropathy [28, 29] and has been reported to have a repressive role in BKPyV replication [30, 31]. In the present study, we demonstrated the localization and expression of JCPyV- and BKPyV-encoded miRNAs in the tissues of patients with virus-associated diseases. The function of JCPyV-miRNA in virus replication was investigated to speculate on the roles of viral miRNAs in disease.

## Results

### Expression of polyomavirus-encoded miRNAs in pathological samples

**Fig 1:**
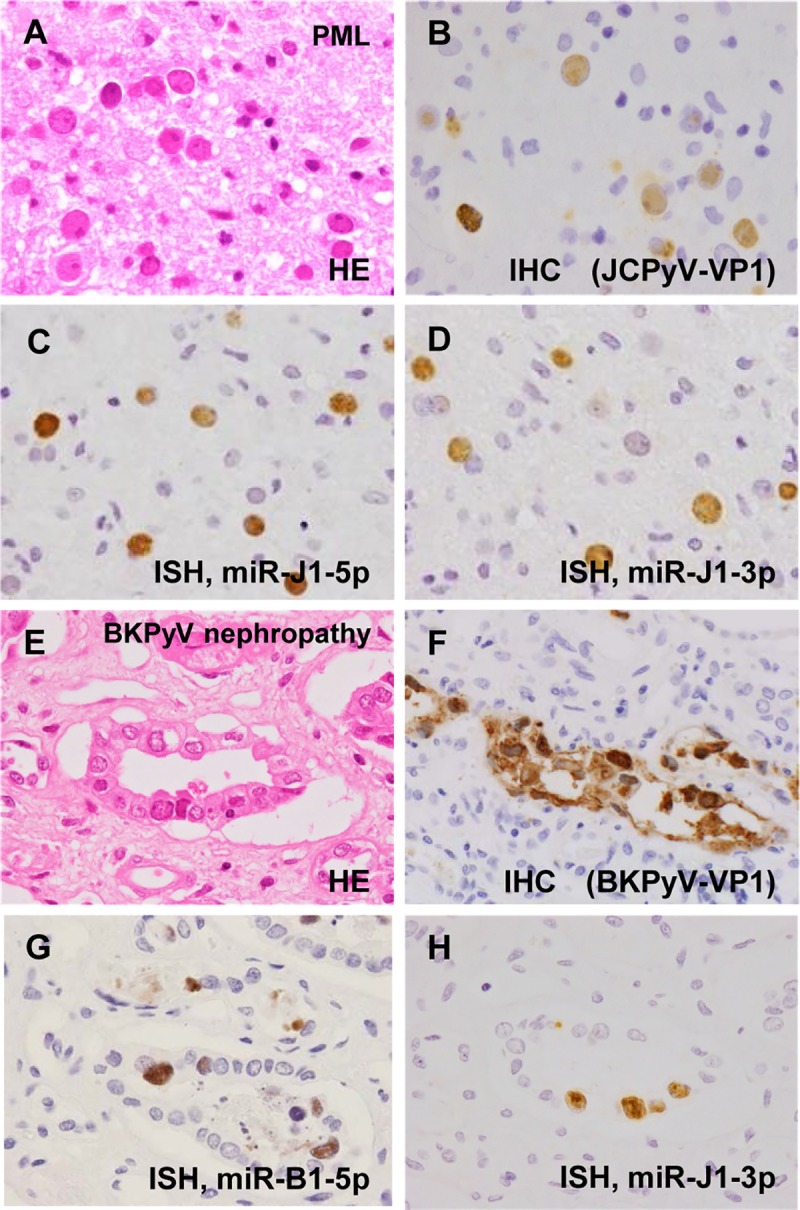
*In situ* hybridization (ISH) for miR-J1 and miR-B1 in clinical samples. (A–D) PML. (A) Haematoxylin and eosin (HE) and (B) immunohistochemistry for JCPyV VP1, (C) ISH for miR-J1-5p, and (D) ISH for miR-J1-3p. (E–H) BKPyV-associated nephropathy. (E) HE and (F) immunohistochemistry for BKPyV VP1, (G) ISH for miR-B1-5p, and (H) ISH for miR-J1-3p.

**Fig 2:**
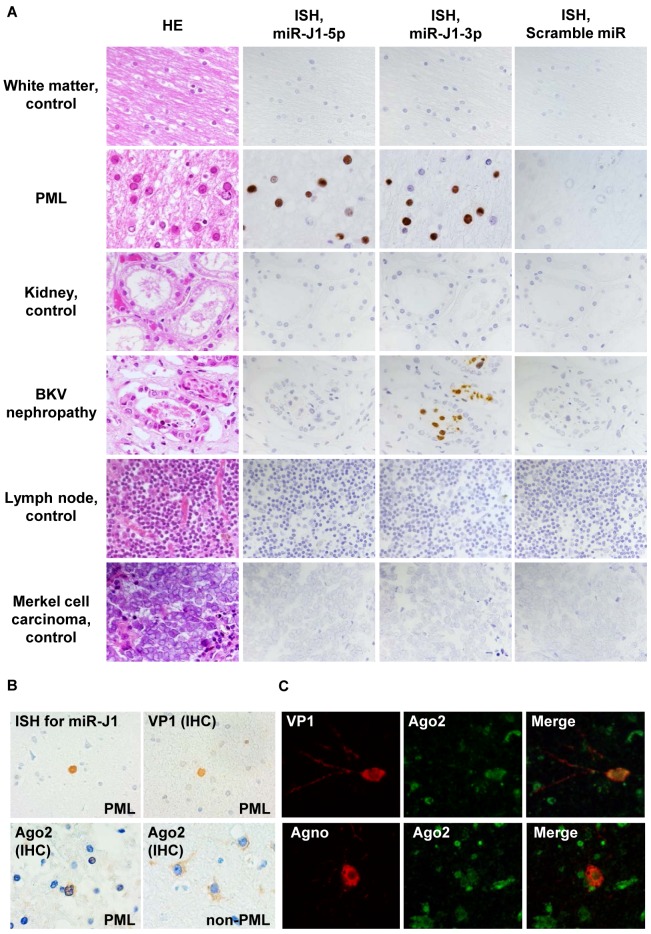
Specificity of *in situ* hybridization (ISH) for miR-J1 and Ago2 expression in clinical samples. (A) ISH for miR-J1 in clinical samples. HE and ISH for miR-J1-5p, miR-J1-3p, and a scramble probe are shown. All control samples were obtained from patients with conditions unrelated to PML or BKPyV-associated nephropathy. (B) miR-J1, JCPyV-VP1, and Ago2 protein expression in a case of PML. *In situ* hybridization detected miR-J1 in the PML case (upper left). JCPyV VP1 (upper right) and Ago2 (lower panels) were detected with immunohistochemistry in a PML lesion (upper right and lower left) and non-PML lesion (lower right). (C) Immunofluorescence assay for JCPyV VP1, agnoprotein, and Ago2 in a case of PML. JCPyV proteins (VP1 and agnoprotein) and Ago2 were labelled with red and green, respectively.

To identify the expression and localization of virus miRNA in PML tissues, we established *in situ* hybridization optimized for detecting viral miRNA. JCPyV-infected oligodendrocytes in PML lesions demonstrated enlarged nuclei. Bizarre astrocytes were often observed in the lesions. *In situ* hybridization revealed that both miR-J1-5p and -3p were expressed in the enlarged nuclei of oligodendrocytes that were positive for JCPyV-encoded VP1 in immunohistochemistry (Fig 1). The miR-J1-3p probe also positively marked the nuclei of renal tubular epithelial cells in BKPyV-associated nephropathy because a BKPyV-encoded miRNA, miR-B1-3p, has the same sequence as miR-J1-3p. miR-B1-5p was detected in BKPyV-associated nephropathy samples with a miR-B1-5p-specific probe, whereas a probe for miR-J1-5p, which has a different sequence from that of miR-B1-5p, did not detect any signal in BKPyV-associated nephropathy (Figs 1 and 2). PML samples, which were confirmed as BKPyV-negative by PCR, showed very weak signals in *in situ* hybridization using a miR-B1-5p probe (Table 1), due to partial high sequence similarity between miR-B1-5p and miR-J1-5p (71%). Probes specific for polyomavirus-encoded miRNAs did not detect any signal in polyomavirus-negative tissues (Fig 2, Table 1). Since mature miRNA forms a complex with Argonaute2 (Ago2) in cells [32], Ago2 expression was examined in the JCPyV-infected cells in a PML lesion. Immunohistochemistry and immunofluorescence assays revealed Ago2 expression in both the nucleus and cytoplasm of JCPyV-infected cells within the PML lesion, whereas Ago2 was detected predominantly in the cytoplasm of the uninfected cells on the same slide (Fig 2B and 2C). These data indicate that *in situ* hybridization specifically detects polyomavirus-encoded miRNAs in tissues. Twenty-five PML tissues, including autopsy and biopsy samples, were examined with *in situ* hybridization. Positive *in situ* hybridization signals were detected for miR-J1-5p and -3p in 24/25 (96%) of the tested PML tissues, indicating comparable sensitivity to immunohistochemistry and PCR (Table 2).

**Table 1:** *In situ* hybridization for polyomavirus miRNA in pathological samples.

Sample	JCV-J1-5p	JCV-J1-3p	BKV-B1-5p	MCV-M1-5p	miR scramble, N.C.
Brain, control	−	−	−	Not tested	−
Kidney, control	−	−	−	Not tested	−
Lymph node, control	−	−	−	−	−
PML brain	+	+	+ (weak)	−	−
BKV nephropathy	−	+	+	Not tested	−
Merkel cell carcinoma	−	−	Not tested	−	−

**Table 2:** Results of *in situ* hybridization (ISH), immunohistochemistry (IHC), and PCR in histological samples.

Autopsy/Biopsy	ISH (miR-J1-5p)	ISH (miR-J1-3p)	IHC (JCV VP1)	JCV DNA-PCR
Autopsy	10/10 (100%)	10/10 (100%)	10/10 (100%)	5/5 (100%)
Biopsy	14/15 (93.3%)	14/15 (93.3%)	15/15 (100%)	14/14 (100%)
Total	24/25 (96.0%)	24/25 (96.0%)	25/25 (100%)	19/19 (100%)

### Quantification of miRNA copies in clinical tissue samples

**Fig 3:**
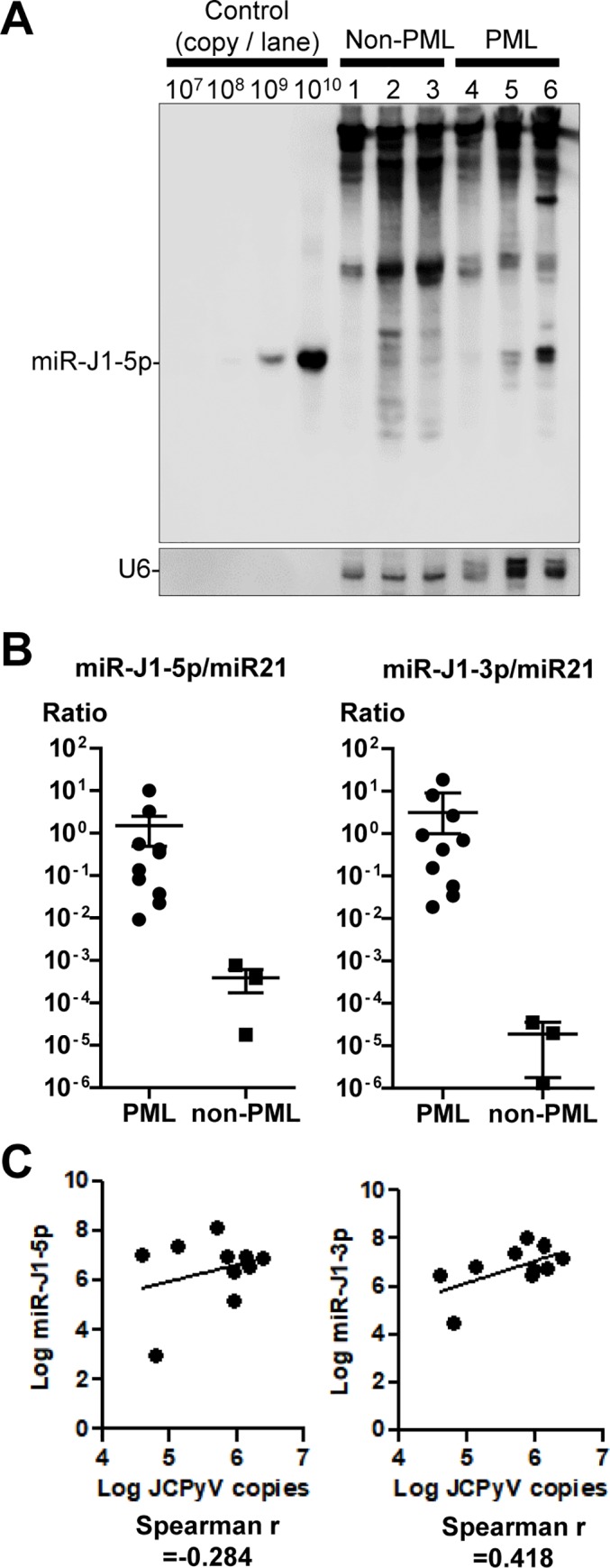
Detection of miR-J1 expression by northern blot and real-time PCR. (A) Northern blot analysis. Three PML and non-PML brain tissues were examined. (B) Stem-loop real-time RT-PCR for miR-J1 in clinical samples from PML (n = 10) and non-PML (n = 3) subjects. The relative ratio of miR-J1/miR21 is shown. Horizontal and vertical bars indicate the mean and standard error, respectively. (C) Correlation between miR-J1 and JCPyV-DNA copy numbers. The numbers of miR-J1 copies per ng of RNA and JCPyV DNA copies per ng of DNA in each sample were plotted on the vertical and horizontal axes, respectively. Ten samples of JCPyV-positive PML tissues were examined. The Spearman’s rank correlation is indicated at the bottom of each graph.

JCPyV-encoded miRNA was also detected with northern blot hybridization (Fig 3A). Although bands of miR-J1-5p were detected in JCPyV-positive cases, non-specific signals were detected in all clinical samples, including RNA extracted from non-PML tissues that were confirmed as JCPyV-negative by PCR; this result is similar to that of a previous report [23]. In addition, semi-quantitative blotting showed that a miRNA copy number of higher than 10^8^ was required to detect a signal (Fig 3A). Thus, northern blot hybridization was not sensitive or specific for detecting the miRNAs. JCPyV-encoded miRNAs were quantified in clinical tissue samples from PML (n = 10) and non-PML (n = 3) subjects with stem-loop real-time reverse transcription (RT)-PCR. miR-J1 was detected not only in the PML samples but also in the non-PML samples by both methods (Fig 3B). When the copy numbers of miR-J1 were standardized by the miR21 copy number, all PML samples showed a higher ratio compared with the non-PML samples (*p* = 0.007 for miR-J1-5p/miR21 and *p* = 0.007 for miR-J1-3p/miR21, Fig 3B). The copy numbers of miR-J1-3p were weakly related to the JCPyV DNA copy numbers in JCPyV-positive samples (Fig 3C).

### Next generation sequencing of small RNAs from polyomavirus-infected samples

**Fig 4:**
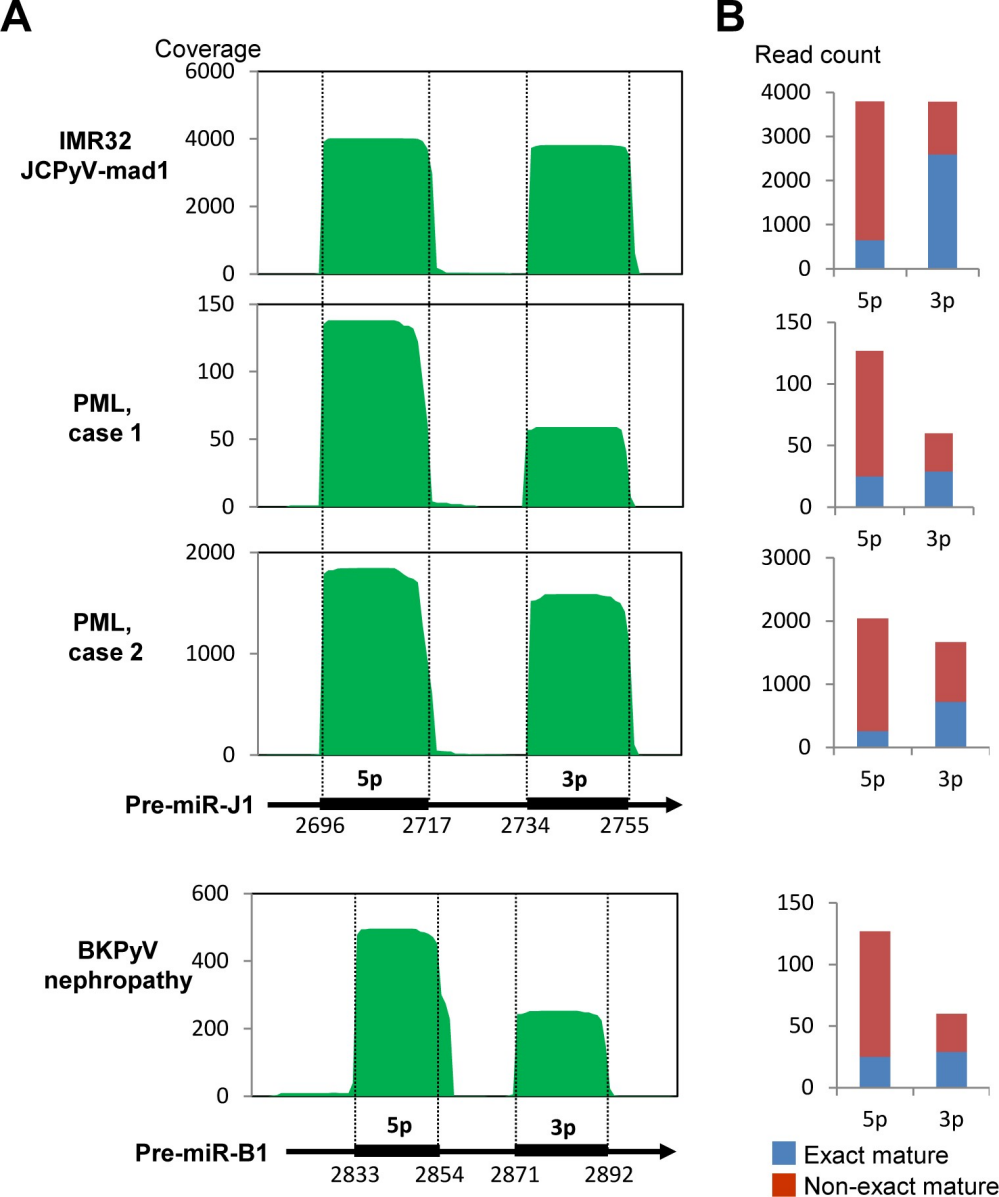
Expression profile of miR-J1 by next generation sequencing. (A) Read coverages of miRNA reads for pre-miR-J1 and miR-B1. Pre-miRNA sequences are shown at the bottom of the graph. Numbers under the bars of 5p and 3p indicate the start and stop nucleotides of 5p and 3p miRNA, respectively, in the reference sequences (JCPyV: J02226 and BKPyV: NC_001538). (B) Read counts of exact and non-exact mature miRNA in each sample.

To determine the miRNA expression profiles in PML and BKPyV-associated nephropathy, small RNAs from polyomavirus-infected samples were sequenced by next generation sequencing (NGS). In all three cases (two PML cases and one BKPyV-associated nephropathy case), 0.1%–4.5% of all annotated miRNAs were derived from polyomavirus (Table 3). RNA extracted from the two PML tissues contained many reads of miR-181a and miR-26a, which are abundantly expressed in neural tissues [33, 34]. The BKPyV-associated nephropathy sample contained the highest number of reads of miR-10, which is common in kidney tissue [35]. Circularized JCPyV Mad1 DNA-transfected IMR32 cells were also examined as a control. miR-J1 made up 7.8% of all annotated miRNAs in the JCPyV-transfected IMR32 cells (Table 3). Polyomavirus-encoded miRNAs were listed in the top 50 miRNAs with high expression in both one PML case and the BKPyV-associated nephropathy case (Table 4). The coverage profile of polyomavirus-encoded miRNAs showed that the pre-miRNA of both miR-J1 and miR-B1 produced both 5p and 3p miRNAs, and that 5p miRNAs were more abundant than 3p miRNAs in each sample (Fig 4). These miRNAs included many non-exact mature miRNAs with an addition or deletion at the 3′ end of miR-J1-5p and miR-B1-5p. 5p miRNAs contained a non-exact mature form of miRNA more frequently compared with 3p miRNA (Fig 4B).

**Table 3:** NGS data from circularized JCPyV Mad1 DNA-transfected IMR32 cells, two PML cases, and one BKV-associated nephropathy case.

	JCV-mad1-IMR32		PML-Case 1		PML-Case 2		BKV-nephropathy	
Annotation	Count	Percentage	Count	Percentage	Count	Percentage	Count	Percentage
Annotated	114,098	72.50%	161,133	77.30%	208,132	38.40%	372,587	48.50%
= - with miRBase	99,463	87.20%	144,900	89.90%	86,404	41.50%	210,211	56.40%
- Homo sapiens	91,719	92.20%	144,709	99.90%	82,485	95.50%	209,439	99.60%
- JC polyomavirus	7,744	7.80%	191	0.10%	3,919	4.50%	-	-
- BK polyomavirus	-	-	-	-	-	-	772	0.40%
= - with Homo_sapiens.GRCh38.ncrna	9,741	8.50%	12,516	7.80%	79,075	38.00%	118,643	31.80%
= - with human-trna	4,894	4.30%	3,717	2.30%	42,653	20.50%	43,733	11.70%
Unannotated	43,350	27.50%	47,208	22.70%	333,216	61.60%	396,219	51.50%
Total	157,448	100.00%	208,341	100.00%	541,348	100.00%	768,806	100.00%

**Table 4:** Top 50 miRNAs in each sample. Polyomavirus-encoded miRNAs are indicated in red.

	JCV-mad1-IMR32			PML-Case 1			PML-Case 2			BKV-nephropathy	
	miRNA	%		miRNA	%		miRNA	%		miRNA	%
**1**	mir-92a-1//mir-92a-2-3p	16.45%		mir-181a-2//mir-181a-1-5p	28.02%		mir-181a-2//mir-181a-1-5p	13.42%		mir-10b-5p	25.97%
**2**	mir-181a-2//mir-181a-1-5p	11.79%		mir-26a-1//mir-26a-2-5p	15.23%		mir-27b-3p	7.73%		mir-143-3p	13.22%
**3**	mir-27b-3p	7.20%		let-7a-1//let-7a-2//let-7a-3-5p	4.26%		mir-26a-1//mir-26a-2-5p	7.59%		mir-10a-5p	10.12%
**4**	mir-10b-5p	6.54%		mir-22-3p	3.21%		mir-21-5p	4.97%		mir-30a-5p	9.94%
**5**	mir-182-5p	4.95%		mir-127-3p	3.09%		mir-22-3p	4.13%		mir-21-5p	6.71%
**6**	mir-J1-5p	3.88%		mir-27b-3p	2.14%		mir-338-3p	3.91%		mir-26a-1//mir-26a-2-5p	5.37%
**7**	mir-J1-3p	3.87%		mir-9-1//mir-9-2//mir-9-3-5p	2.05%		mir-9-1//mir-9-2//mir-9-3-5p	3.19%		mir-27b-3p	2.54%
**8**	let-7a-1//let-7a-2//let-7a-3-5p	3.51%		mir-191-5p	1.80%		mir-J1-5p	2.58%		mir-126-5p	1.77%
**9**	mir-181b-1//mir-181b-2-5p	2.63%		mir-30d-5p	1.57%		mir-143-3p	2.54%		let-7f-1//let-7f-2-5p	1.48%
**10**	mir-26a-1//mir-26a-2-5p	2.45%		mir-30a-5p	1.57%		let-7a-1//let-7a-2//let-7a-3-5p	2.49%		let-7i-5p	1.44%
**11**	mir-16-1//mir-16-2-5p	2.43%		mir-125b-1//mir-125b-2-5p	1.56%		mir-181b-1//mir-181b-2-5p	2.16%		mir-192-5p	1.38%
**12**	mir-25-3p	2.00%		mir-92b-3p	1.50%		mir-30a-5p	2.12%		mir-22-3p	1.29%
**13**	mir-30d-5p	1.82%		let-7b-5p	1.47%		mir-J1-3p	2.11%		mir-181a-2//mir-181a-1-5p	1.26%
**14**	mir-92b-3p	1.77%		let-7c-5p	1.32%		mir-100-5p	2.02%		mir-199a-1//mir-199a-2//mir-199b-3p	0.91%
**15**	mir-191-5p	1.48%		mir-181b-1//mir-181b-2-5p	1.30%		let-7f-1//let-7f-2-5p	1.99%		mir-101-1//mir-101-2-3p	0.88%
**16**	mir-22-3p	1.35%		mir-143-3p	1.27%		mir-125b-1//mir-125b-2-5p	1.48%		let-7a-1//let-7a-2//let-7a-3-5p	0.76%
**17**	mir-143-3p	1.09%		mir-486-1//mir-486-2-5p	1.23%		let-7i-5p	1.46%		mir-146b-5p	0.65%
**18**	mir-151a-3p	1.03%		mir-769-5p	1.13%		mir-219a-1//mir-219a-2-5p	1.39%		mir-30e-5p	0.64%
**19**	mir-103a-2//mir-103a-1-3p	0.99%		mir-92a-1//mir-92a-2-3p	1.12%		mir-127-3p	1.37%		mir-26b-5p	0.59%
**20**	mir-186-5p	0.96%		mir-103a-2//mir-103a-1-3p	1.12%		mir-126-5p	1.28%		mir-30d-5p	0.55%
**21**	mir-99b-5p	0.78%		let-7f-1//let-7f-2-5p	1.11%		mir-16-1//mir-16-2-5p	1.21%		mir-92a-1//mir-92a-2-3p	0.53%
**22**	mir-93-5p	0.74%		mir-128-1//mir-128-2-3p	1.01%		mir-30e-5p	1.13%		mir-148a-3p	0.45%
**23**	mir-21-5p	0.59%		mir-125a-5p	1.00%		mir-191-5p	1.12%		mir-16-1//mir-16-2-5p	0.42%
**24**	mir-127-3p	0.56%		mir-181c-5p	0.92%		mir-101-1//mir-101-2-3p	1.12%		mir-191-5p	0.42%
**25**	mir-181a-1-3p	0.54%		mir-99b-5p	0.73%		mir-29a-3p	1.11%		let-7g-5p	0.39%
**26**	mir-199a-1//mir-199a-2//mir-199b-3p	0.50%		mir-151a-3p	0.71%		mir-4454-5p	1.06%		mir-126-3p	0.33%
**27**	mir-378a-3p	0.48%		mir-30c-2//mir-30c-1-5p	0.61%		mir-146b-5p	0.90%		mir-141-3p	0.32%
**28**	mir-125a-5p	0.48%		let-7g-5p	0.54%		mir-92a-1//mir-92a-2-3p	0.85%		mir-186-5p	0.30%
**29**	let-7f-1//let-7f-2-5p	0.47%		mir-451a-5p	0.54%		mir-103a-2//mir-103a-1-3p	0.81%		mir-151a-3p	0.27%
**30**	let-7i-5p	0.47%		mir-151a-5p	0.53%		mir-26b-5p	0.74%		mir-103a-2//mir-103a-1-3p	0.26%
**31**	mir-30e-5p	0.46%		mir-100-5p	0.49%		mir-99b-5p	0.74%		mir-99b-5p	0.26%
**32**	let-7g-5p	0.46%		mir-29a-3p	0.47%		mir-99a-5p	0.69%		mir-29a-3p	0.25%
**33**	mir-9-1//mir-9-2//mir-9-3-5p	0.42%		mir-149-5p	0.47%		mir-186-5p	0.66%		mir-19b-1//mir-19b-2-3p	0.25%
**34**	mir-28-3p	0.41%		mir-16-1//mir-16-2-5p	0.45%		mir-151a-5p	0.60%		mir-25-3p	0.24%
**35**	mir-17-5p	0.40%		let-7i-5p	0.42%		let-7c-5p	0.58%		mir-30a-3p	0.24%
**36**	mir-151a-5p	0.39%		mir-221-3p	0.38%		mir-219a-2-3p	0.56%		mir-B1-5p	0.23%
**37**	mir-423-3p	0.33%		mir-126-5p	0.36%		mir-92b-3p	0.55%		mir-130a-3p	0.22%
**38**	mir-20a-5p	0.32%		mir-21-5p	0.35%		mir-125a-5p	0.55%		let-7b-5p	0.21%
**39**	let-7b-5p	0.32%		mir-410-3p	0.35%		let-7b-5p	0.54%		mir-100-5p	0.19%
**40**	mir-301a-3p	0.31%		mir-204-5p	0.33%		mir-29c-3p	0.53%		mir-125a-5p	0.18%
**41**	mir-130a-3p	0.31%		mir-124-1//mir-124-2//mir-124-3-3p	0.28%		mir-124-1//mir-124-2//mir-124-3-3p	0.53%		mir-28-3p	0.18%
**42**	mir-30c-2//mir-30c-1-5p	0.29%		mir-30e-5p	0.28%		let-7g-5p	0.51%		mir-125b-1//mir-125b-2-5p	0.18%
**43**	mir-363-3p	0.29%		mir-101-1//mir-101-2-3p	0.28%		mir-151a-3p	0.49%		mir-30c-2//mir-30c-1-5p	0.17%
**44**	mir-340-5p	0.28%		mir-146b-5p	0.28%		mir-30d-5p	0.48%		mir-98-5p	0.17%
**45**	mir-183-5p	0.28%		let-7e-5p	0.27%		mir-25-3p	0.45%		mir-21-3p	0.17%
**46**	mir-101-1//mir-101-2-3p	0.28%		mir-28-3p	0.26%		mir-128-1//mir-128-2-3p	0.44%		mir-29c-3p	0.16%
**47**	mir-148a-3p	0.26%		mir-219a-2-3p	0.26%		mir-181c-5p	0.40%		mir-378a-3p	0.15%
**48**	mir-30b-5p	0.26%		mir-138-2//mir-138-1-5p	0.26%		mir-769-5p	0.38%		mir-27a-3p	0.15%
**49**	mir-125b-1//mir-125b-2-5p	0.24%		mir-186-5p	0.26%		mir-340-5p	0.35%		mir-204-5p	0.15%
**50**	mir-192-5p	0.24%		mir-30b-5p	0.26%		mir-30c-2//mir-30c-1-5p	0.31%		mir-142-5p	0.15%
			80	mir-J1-5p	0.09%				59	mir-B1-3p	0.12%
** **			114	mir-J1-3p	0.04%						

### Repressive function of miR-J1

**Fig 5:**
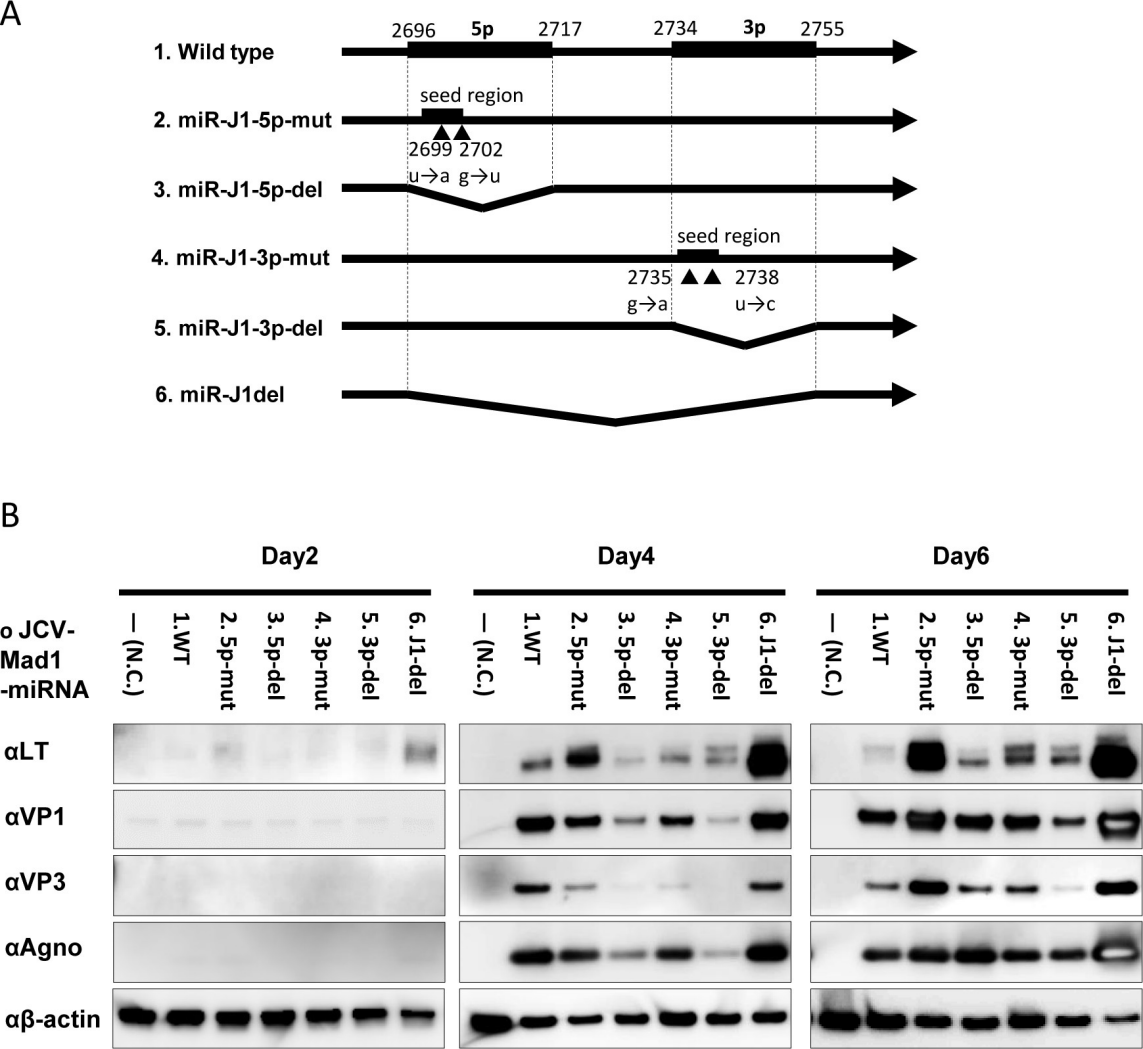
JCPyV replication in miR-J1-mutants. (A) Construction of JCPyV mutants. Point or deletion mutants were constructed in pre-miR-J1 of the JCPyV genome. (B) Western blot analysis on lysates of mutant-transfected IMR32 cells. Each number (1–6) corresponds to that in (A). The results from samples taken on day 2, 4, and 6 post-transfection are shown.

Mutant JCPyV DNAs were constructed to investigate the roles of miR-J1 in a recombinant JCPyV-replication system (Fig 5A) [36]. With wildtype miR-J1, LT expression was low on day 2 post-transfection, increased on day 4, and decreased on day 6. Mutations without amino acid changes in the seed regions of miR-J1-5p and -3p resulted in higher expression of the LT compared with wildtype JCPyV on day 6 post-transfection (Fig 5B). Deletion of the whole miR-J1 sequence, miR-J1-5p, or miR-J1-3p also induced higher expression of LT on day 6 post-transfection. Furthermore, a deletion mutant of the whole miR-J1 sequence showed higher expression of late viral proteins, such VP1, -3, and agnoprotein, compared with wildtype JCPyV. These data suggest that miR-J1 plays a repressive role in the replication of JCPyV.

## Discussion

Here, we used *in situ* hybridization to show the expression and localization of virus-encoded miRNA in polyomavirus-associated diseases. To our knowledge, this is the first report showing high expression and clear localization of virus-encoded miRNA in virus-associated disease tissues by *in situ* hybridization. Although several previous reports have described the *in situ* detection of viral miRNAs such as HSV2 [37], EBV [38, 39], and HPV [40], no clear evidence of miRNA localization in tissue samples has been demonstrated.

Its nuclear localization provides insight into the likely function of the miRNA in virus replication. The localization of cellular miRNAs varies depending on the miRNA and cell types. For example, in neurons, miR155 and miR21 are expressed predominantly in the nuclei [41, 42], whereas in lymphoma cells, these miRNA are rarely expressed in the nuclei but are found abundantly in the cytoplasm [43]. JCPyV replicates and accumulates mainly in the nuclei of virus-infected cells within PML lesions [44]. The accumulation of a large amount of virus results in enlarged nuclei [45]. JCPyV-encoded LT and VP1-3 are expressed in the nucleus, whereas agnoprotein is expressed in the cytoplasm [45]. Generally, miRNA binds to transcripts to regulate protein expression [1, 2]. Considering the repressive role of JCPyV miRNA in viral replication, nuclear localization seems to be effective for binding and suppressing the translation of these virus-encoded mRNAs.

A repressive function of miR-J1 on LT expression was previously shown in a reporter assay using a plasmid containing the genomic region of miR-J1 pre-miRNA in LT [23]. Our study clearly showed a repressive effect of miR-J1 on JCPyV replication using transfection with miR-J1-deletion or miR-J1-mutant viral genomes. Complete deletion of the miR-J1 sequence and individual deletion of miR-J1-5p or -3p both resulted in high viral protein expression (Fig 5), implying that miR-J1 may repress JCPyV replication in JCPyV-infected cells. The existence of a weak relationship between the copies of miR-J1-3p and JCPyV DNA in PML lesions identified by quantitative analysis (Fig 3C) suggests that miR-J1 is effectively produced in the replication stage of JCPyV. In contrast, an inverted relationship was previously reported between miR-J1 and JCPyV DNA in blood and cerebrospinal fluid [24]. Taken together, these findings suggest that miR-J1 contributes to reducing the amount of JCPyV DNA in the serum and cerebrospinal fluid, but the amount of miR-J1 may not be sufficient to substantially reduce the number of JCPyV copies in PML lesions. Our previous study demonstrated that JCPyV replicates in the presence of a mutant virus that can repress virus replication in PML lesions [36]. JCPyV-encoded miRNA, therefore, may be another repressive function of JCPyV itself. The resolution of such a repressive mechanism could lead to the development of new treatments for PML or JCPyV infection.

JCPyV infection is an important criterion for PML diagnosis [22]. Immunohistochemistry detecting JCPyV-encoded proteins has been used for pathological diagnosis [45, 46]. Although the number of samples analyzed in this study (25 samples) was small, the *in situ* hybridization results for JCPyV miRNA showed comparable sensitivity to immunohistochemistry (Table 2). While northern blot and real-time PCR showed non-specific reactions even in the non-PML tissues, *in situ* hybridization for viral miRNA demonstrated clear signals in JCPyV-infected cells without any non-specific signals (Figs 1 and 2). Quantitative study and NGS demonstrated high copy numbers of both miR-J1-5p and -3p in PML lesions, suggesting that the identification of these miRNAs by *in situ* hybridization may be a possible marker for JCPyV infection. In terms of specificity, the miR-J1-5p probe used in the present study did not cross-react with BKPyV miRNA (Fig 2); thus, it has potential to be used for differential diagnosis between JCPyV and BKPyV infection. *In situ* hybridization for miR-J1 and miR-B1 will be a useful tool in the diagnosis of JCPyV and BKPyV infections. Because miRNA is a small molecule of nucleotides, miRNA is rarely fragmented and is conserved in formalin-fixed and paraffin-embedded (FFPE) samples, unlike mRNAs [47], so the evaluation of miRNA should be possible even from FFPE tissues remaining from past autopsy. The *in-situ* detection of viral miRNA from surgical specimens will provide pathologists with a novel strategy for the diagnosis of viral infectious diseases.

In the NGS results, non-exact mature miRNAs were included in all samples at high frequencies. This phenomenon has been previously observed for other virus and mammalian miRNAs as well [48]. Interestingly, miR-J1-5p contained non-exact mature miRNAs at a higher frequency compared with miR-J1-3p. It has been reported that the expression ratio of exact and non-exact mature miRNAs varies among miRNAs [49]. In addition, non-exact mature miRNA is detected more frequently in extracellular vesicles (exosomes) than in intracellular components [49, 50]. Although no difference of function between exact and non-exact mature miRNAs has been reported, the detection rate differed between the two PCR systems used (the miScript PCR system and stem-loop real-time RT-PCR) [48]. Further studies will be required to reveal the expression mechanism and function of such non-exact mature miRNAs in virus infection.

In conclusion, high expression and nuclear localization of polyomavirus-encoded miRNAs were demonstrated in tissues from PML and BKPyV-associated nephropathy cases by *in situ* hybridization. The sensitivity of *in situ* hybridization for viral miRNA was comparable to that of immunohistochemistry. Therefore, *in situ* hybridization for viral miRNA may be a useful diagnostic tool for PML. Additionally, the deletion of miR-J1 in JCPyV DNA resulted in high expression of viral proteins in JCPyV DNA-transfected cells, implying a repressive function for miR-J1.

## Materials and methods

### Ethics Statement

All procedures in studies involving human tissues were performed in accordance with the ethical standards of the Institutional Review Board of the National Institute of Infectious Diseases (Approval No. 595) and those of the 1964 Helsinki declaration and its later amendments or comparable ethical standards.

### Clinical samples

All clinical samples were collected from Japanese patients in Japan. All data in the present study were analyzed anonymously. Brain tissue samples from 32 patients with PML (male = 71.8%, mean age = 55.7 ± 14.8 years), including 11 autopsy and 21 biopsy samples, and four normal control individuals were examined. The background conditions of the 32 patients with PML were: AIDS (11 cases), hematologic malignancy (8 cases), autoimmune diseases (7 cases), organ transplantation (2 cases), congenital immunodeficiency (1 case), and unknown (3 cases). A kidney tissue sample from a patient with BKPyV-associated nephropathy was also examined. All tissue samples were originally submitted from hospitals or institutes to the Department of Pathology at the National Institute of Infectious Diseases for diagnostic consultation, and all the samples used in this study were residual tissues remaining after the diagnostic purpose for which they had been collected was fulfilled. For NGS experiments, we examined two brain samples of PML cases and one kidney sample from a BKPyV-associated nephropathy case. All PML cases were histologically confirmed to have PML and confirmed to be positive for JCPyV infection by PCR for JCPyV-DNA and/or immunohistochemistry. In addition to these samples, one case of Merkel cell carcinoma and kidney and lymph node tissues from unrelated patients without any polyomavirus-associated diseases were examined as controls.

### Immunohistochemistry and immunofluorescence assay

Immunohistochemistry was performed as described previously [36]. Anti-JCPyV VP1 (rabbit polyclonal [51]), agnoprotein (rabbit polyclonal [45]), BKPyV-VP1 (mouse monoclonal, Abnova, Taipei, Taiwan), or Ago2 (Clone 2D4, mouse monoclonal, Fujifilm Wako, Osaka, Japan) antibody was used as the primary antibody. In the immunofluorescence assays, Alexa 568-conjugated anti-rabbit IgG antibody and Alexa 488-conjugated anti-mouse IgG antibody were used as the secondary antibodies.

### *In situ* hybridization

*In situ* hybridization for miRNA was performed using microRNA ISH Buffer Set (Exiqon no. 90000, Qiagen, Hilden, Germany) on FFPE samples as described previously [52]. Briefly, deparaffined slides were washed three times with tris-buffered saline (TBS), followed by treatment with 0.3% H_2_O_2_/methanol at room temperature (RT) for 30 min. The slides were then incubated for 20 min with 3 μg/ml (for biopsy samples) or 10 μg/ml (for autopsy samples) of proteinase K at 37°C. After being washed with TBS and dehydrated, the slides were hybridized for 2 h (for biopsy samples) or overnight (for autopsy samples) with a specific probe (50-nM concentration) at 55°C. The slides were washed with 2× SSC for 15 min at RT twice, then with 0.2 × SSC for 15 min at 50°C twice. After being washed with TBS, the slides were incubated with anti-digoxigenin biotin-conjugated mouse monoclonal antibody (B-7405, 1,000× dilution, Sigma-Aldrich, St. Louis, MO, USA) at RT for 60 min. The slides were serially incubated with GenPoint kit reagents (Dako, Copenhagen, Denmark): first with a primary antibody of streptavidin–horseradish peroxidase (2,000× dilution) at RT for 20 min, followed by biotintylamide at RT for 15 min, and last with the secondary antibody streptavidin–horseradish peroxidase at RT for 15 min. Finally, the signals were visualized by 3,3′-diaminobenzidine, and the slides were counterstained, dehydrated, and mounted. The probes used were JCV-miR-J1-5p, JCV-miR-J1-3p, BKV-miR-B1-5p, MCV-miR-M1-5p, and miR-scramble probes (miRCURY LNA Detection probe, 250 pmol, 5′-DIG and 3′-DIG labelled, Exiqon).

### RNA extraction

Total RNA, including miRNA, was extracted from frozen or paraffin-embedded samples and cultured cells using the Isogen (Nippon Gene, Tokyo, Japan) or High Pure miRNA purification kits (Roche Molecular Biochemicals, Indianapolis, IN, USA) and subsequently treated with Turbo DNase (Ambion, Austin, TX, USA), in accordance with the instructions from the manufacturers.

### Northern blot for miRNA

RNAs were extracted from PML and non-PML brain tissues. Northern blotting analysis for miRNA was performed as described previously [53]. JCV-miR-J1-5p and U6 probes (miRCURY LNA Detection probe, 5′-DIG and 3′-DIG labelled, Exiqon, Qiagen) were used as detection probes. Synthesized miR-J1 RNA was serially diluted and examined as a positive control.

### Real-time PCR for JCPyV DNA in tissue samples

DNA was extracted from FFPE samples or frozen tissues with QIAamp DNA FFPE Tissue Kit (Qiagen) or DNeasy Blood and Tissue Kit (Qiagen), respectively. JCPyV DNA was quantified with TaqMan real-time PCR, as described previously [54].

### Real-time RT-PCR for JCPyV and BKPyV miRNA

Real-time RT-PCR for the quantification of miR-J1-5p, miR-J1-3p, and the human cellular miRNA miR21-5p was carried out with stem-loop real-time RT-PCR (TaqMan Small RNA assay, Applied Biosystems, Foster City, CA, USA), in accordance with the instructions from the manufacturers. Each reaction was carried out in triplicate with 10 ng of RNA and included no template controls. Synthesized oligonucleotides of miR-J1-5p, miR-J1-3p, and miR21-5p were used as standards. Ratios of the copy numbers of virus-encoded miRNA to miR21 were calculated as follows: ratio of target miRNA to miR21 = 2^Ct of miR21^/2^Ct of target^ (Ct = cycle threshold).

### Next generation sequencing (NGS)

Small RNA libraries were established with the TruSeq small RNA kit (Illumina, San Diego, CA, USA) from 18- to 35-nucleotide cDNAs using 5 μg of DNase-treated total RNA. Small RNA was sequenced using the MiSeq (Illumina) with MiSeq reagent kit v3. Sequence reads were analyzed with CLC Genomics Workbench (version 12.0.3, Qiagen). After adaptor trimming, reads of less than 15 or more than 26 nucleotides in length were removed, and all reads of 15–25 nucleotides in length were analyzed against miRBase release 21 retrieved from the miRNA database (http://www.mirbase.org/). Homo_sapiens.GRCh38.ncrna was used as a comprehensive noncoding RNA database (http://www.ncrna.org/). Human tRNA was annotated using the GRCh38/hg38 tRNA database (GtRNAdb, http://gtrnadb.ucsc.edu/). Reads matching pre-miRNA were counted as miRNA reads. The ratio of the read numbers of mature miRNAs to the total annotated miRNAs was analyzed between the samples.

### Cell culture

The human neuroblastoma cell line IMR-32 was purchased from the Health Science Research Resource Bank (Osaka, Japan). IMR-32 cells were cultured in Dulbecco’s Modified Eagle’s Medium (DMEM, Thermo Fisher Scientific, Rockford, IL, USA) with 10% fetal bovine serum (FBS), 0.1 mM MEM non-essential amino acids (Thermo Fisher Scientific), penicillin, and streptomycin (Thermo Fisher Scientific).

### JCPyV genome recombination experiments

The complete genome of the JCPyV Mad1 strain (GenBank J02226), with the insertion of GGTC between nucleotide positions 109 and 110, was subcloned into pUC19. Mutagenesis to construct the JCPyV Mad1 genome was performed with the KOD-Plus-Mutagenesis Kit (Toyobo, Osaka, Japan) following the manufacturer’s protocol. The sequences of the primers were as follows: forward (5′ to 3′) miRJ1-5p-mut-f (ttcagatacctgggaaaagcattgtgattgtg) and reverse (5′ to 3′) miRJ1-5p-mut-r (gcctctggtgcagacacacaggaaaactgc) for miR-J1-5p-mut; miRJ1-3p-mut-f (tactcgatccatgtccagagtcttctgctt) and miRJ1-3p-mut-r (tgaatcacaatcacaatgcttttcccagg) for miRJ1-3p-mut; miRJ1-5p-del-f (tgtgattgtgattcagtgcttgatccatgt) and miRJ1-5p-mut-r for jcv-miR-J1-5p deletion; miRJ1-3p-del-f (ttctgcttcagaatcttcctctctaggaaa) and miRJ1-3p-mut-r for jcv-miR-J1-3p deletion; and miRJ1-5p-mut-r and miRJ1-3p-del-f for jcv-miR-J1-premiRNA deletion. After amplification, the plasmid was digested and self-ligated to construct a complete circular JCPyV Mad1 genome. The mutated circular JCPyV Mad1 genomes were fully sequenced to confirm that all the sequences were identical to the wildtype JCPyV genome except for the altered nucleotide.

### Transfection with viral genomes and immunoblotting

Circular JCPyV DNA was transfected into IMR32 cells as described previously [36]. Briefly, IMR32 cells were seeded onto type I collagen-coated 24-well plates and transfected with 200 ng of viral genome using Attractene Transfection Reagent (Qiagen) in accordance with the manufacturer’s instructions. One day after transfection with viral genomes, the transfected cells were transferred to type I collagen-coated six-well plates and cultured for an additional 1–5 days before collection. Immunoblotting was performed as described previously [36].

### Data deposition

The annotated miRNAs detected by NGS in the clinical samples and cells in this study were deposited in the DNA Data Bank Japan (DDBJ; accession number DRA009067, BioProject PRJDB8864).

### Statistical analysis

The Mann–Whitney U-test was used for nonparametric two group comparison (Graph Pad Prism 5, GraphPad Software, La Jolla, CA, USA).
